# Portopulmonary hypertension

**DOI:** 10.4103/1817-1737.58953

**Published:** 2010

**Authors:** Sarfraz Saleemi

**Affiliations:** *King Specialist Hospital and Research Center, Riyadh 11211, Saudi Arabia*

**Keywords:** Liver transplant, portopulmonary hypertension, pulmonary arterial hypertension, portal hypertension, vasodilator therapy

## Abstract

Portopulmoanry hypertension (POPH) is a form of pulmonary arterial hypertension (PAH) associated with portal hypertension with or without underlying chronic liver disease. POPH is increasingly recognized and recent evidence suggests that it is one of the leading causes of PAH. The pathophysiology of POPH is poorly understood although the pathological changes in pulmonary vasculature in advanced POPH are similar to those seen in idiopathic pulmonary hypertension. The prognosis in patients with liver disease who also suffer from significant POPH is considered to be poor. Higher degree of pulmonary artery pressure (PAP) may preclude a patient from liver transplant as mortality in these patients is high. The treatment with vasodilator therapy has shown to improve both hemodynamics and clinical outcome in POPH in retrospective studies and in some case series. The aim of medical management is to bring PAP <35 mmHg that may make a patient with POPH and advanced liver disease eligible for liver transplant, which otherwise would have been denied because of high PAP.

Portopulmonary hypertension (POPH) is defined as pulmonary arterial hypertension (PAH) complicated by portal hypertension, with or without advanced hepatic disease. It is classified as group 1 in current classification of pulmonary hypertension. Although it affects only 2–5% of the population suffering from portal hypertension, its clinical implications are enormous.[1–3] The prevalence of POPH in patients undergoing liver transplant (LT) is considered to be higher, with one study showing a prevalence of 8.5%.[4] Recent evidence from France shows that POPH is the fourth most common form of PAH reported overall in the population-based French National Registry, after idiopathic PAH and PAH associated with connective tissue diseases and congenital heart disease.[5]

The outcome of LT in the presence of POPH is poor with a 35% reported mortality rate in LT recipients having a mean pulmonary artery pressure (mPAP) >35 mmHg. A patient with significant pulmonary artery pressure (PAP) may be denied the opportunity for transplant unless the mPAP is brought below 35 mmHg with medical treatment. In patients who do undergo successful LT, there can be resolution of pulmonary hypertension with time.

Patients who present with portal hypertension and have an increased mPAP >25 mmHg at rest, elevated pulmonary vascular resistance (PVR) >240 dyne/sec/cm^−5^ and a normal or decreased pulmonary artery wedge pressure (PAWP) < 15 mmHg are considered to have POPH according to the European Respiratory Society Pulmonary Hepatic Vascular Disorder Task Force 2004 Consensus Report [Table 1].

**Table 1:** Diagnostic criteria for portopulmonary hypertension

Clinical portal hypertension with or without significant chronic liver disease
Mean pulmonary artery pressure (mPAP) >25 mmHg at rest
Mean pulmonary capillary wedge pressure (mPCWP) <15 mmHg
Pulmonary vascular resistance (PVR) >240 dyne/sec/cm^−5^ (3.0 Wood Units)

Approximately 30–50% of the patients with cirrhotic liver disease have low systemic vascular resistance and high cardiac output. In these patients, pulmonary arterial pressures may be elevated due to increased cardiac output. These patients have lower values of PVR. Some investigators propose that a cutoff of PVR >120 dynes/s/cm^−5^ can be used in order to define POPH in the presence of hyperdynamic circulatory state.[6] Elevated PVR, despite high cardiac output is one of the unique characteristics of POPH.

Certain risk factors are found to be associated with development of POPH. Female sex and autoimmune hepatitis were associated with an increased risk of POPH, while patients with hepatitis C infection had a lower risk.[7] In addition, genetic variation in estrogen signaling and cell growth regulators is associated with the risk of POPH.[8]

The survival of untreated patients with POPH is very poor. In a recent retrospective study from the Mayo Clinic Liver Transplantation Group, they identified 74 POPH patients from 1994 through 2007, and categorized them into three groups: 1) no therapy for POPH or LT; 2) therapy for POPH alone; and 3) therapy for POPH followed by LT. Five-year survival in 19 patients who received no therapy for POPH and no LT representing the natural history of POPH was 14%, with 54% dying within one year of diagnosis. The median survival in 43 of the 74 patients (58%) who received medical therapy for POPH and did not undergo LT was 46 months and five-year survival was 45%, being significantly better than the patients who were not selected to receive medical therapy for POPH (*P* = 0.03). Twelve patients underwent LT and five-year survival for the nine patients receiving therapy for POPH was 67% compared to 25% in three patients who were not pretreated with prostacyclin therapy.[9]

## Pathogenesis

It has been found that the development of POPH is independent of the cause of the portal hypertension. The severity of the underlying liver disease does not appear to correlate with the severity of POPH.[10] Hyperdynamic circulatory state and high cardiac output are the hallmarks in most of the patients with POPH leading to increased shear stress on the pulmonary circulation. The PVR then rises owing to vasoconstriction, progressive pulmonary vascular remodeling, and *in situ* thrombosis.[11] The histological abnormalities in POPH are almost the same as in idiopathic PAH. The main pathological abnormalities include proliferate arteriopathy, obliteration of the vascular lumen by endothelial and smooth-muscle cells, formation of plexiform lesions, necrotizing arteritis, fibrinoid necrosis and in-situ thrombi.[12–14] The presence of portosystemic shunts may allow the shunting of the vasoactive substances including endothelin 1 (ET-1), vasoactive intestinal peptide, serotonin, thromboxane A2, interleukin 1, glucagon, and secretin from the splanchnic circulation to the pulmonary circulation, allowing these vasoactive mediators to bypass the liver metabolism and causing substantial effects on the pulmonary vasculature.[15,16]

## Clinical Features

Dyspnea on exertion is the most common symptom. Other symptoms include fatigue, generalized weakness, light-headedness, and orthopnea. Physical examination may show abnormalities include accentuated and split second sound, systolic murmur, right ventricular heave, right sided S3 gallop, jugular vein extension, edema and the signs of either decompensated cirrhosis or overt right- heart failure such as ascites and lower leg edema. Lower leg edema out of proportion to ascites due to portal hypertension may suggest associated POPH.[17,18] Arterial blood gas may show hypoxemia and increased A-a gradient. The decrease in arterial oxygenation was found to be significantly worse in patients with POPH when compared with a cohort of patients that underwent screening for LT and normal right ventricular systolic pressures.[19] Electrocardiogram may show evidence of right ventricular hypertrophy, right atrial enlargement and right axis deviation. Chest X-ray is usually normal but may show enlarged pulmonary arteries. Pulmonary function tests may be normal or may show decreased diffusion.

## Diagnosis

According to the guidelines by American Association for the Study of Liver Disease (AASLD), all patients being screened for LT should be evaluated for POPH by transthoracic echocardiography (TTE).[20] In a large prospective POPH screening study (N = 1,235), right ventricular systolic pressure (RVSP) of >50 mmHg was noted in 10.9% of patients. Right heart catheterization was conducted in this group of patients. The diagnosis of POPH based upon the current diagnostic criteria was made in 65% of patients having RVSP >50 mm on TTE. Therefore, 35% of these had “false positives” based solely upon Doppler echocardiography results.[21]

After initial screening with TTE, definitive diagnosis should be made by right heart catheterization that includes measurements of mPAP, PAOP, cardiac output (CO), and calculated PVR.[22] Acute vasoreactivity test with either Nitric Oxide or Prostacyclin can be done not to determine patient selection for calcium channel blocker therapy but to assess prognosis. It is likely that right heart function and other variables play an important role in patient outcome.[23]

The diagnosis of POPH by right heart catheterization before LT is crucial as data suggest an increased risk of death following LT in patients with POPH if the mPAP exceeds 35 mmHg. It is suggested to consider specific vasomodulating treatment for POPH if pre-liver transplant mPAP is greater than 35 mmHg. Patients with mPAP of less than 35 mmHg can be transplanted without undergoing vasodilator treatment for POPH.[24–27]

Right heart catheterization is also important to help classify severity of POPH[28] and therapeutic options based on severity of disease [Table 2].

**Table 2:** Staging of severity of POPH

Variable	Normal	Mild	Moderate	Severe
NYHA Class	-	I, II	II, III	II, IV
mPAP (mmHg)	15–24	25–34	35–44	>45
CI (L/min^−1^/m^2^)	2.5–4	>2.5	>2.5	<2.0
PVR (dynes/s/cm^−5^)	<240	240–500	500–800	>800
RAP (mmHg)	0–5	0–5	5–10	>10
Prognosis	1	Favorable	Questionable	Poor
Specific therapy	-	No	Questionable	Yes
Reversibility after LT	-	Yes	Questionable	No

NYHA = New York Heart Association; mPAP = Mean pulmonary arterial pressure; CI = Cardiac index; PVR = Pulmonary vascular resistance; RAP= Right atrial pressure; OLT = Orthotropic liver transplantation, POPH = Portopulmoanry hypertension

## Management of POPH

### General measures

In addition to the general psychosocial issues, keeping optimum oxygen saturation is crucial. Hypoxemia may worsen POPH through pulmonary vasoconstriction, and therefore supplemental oxygen should be considered for all patients with hypoxemia to maintain oxygen saturation higher than 90% at all times.

### Diuretics

Diuretics have to be used with caution in patients with POPH. Although they are useful in reducing the increased intravascular volume commonly present in chronic liver disease, they can reduce the cardiac output by decreasing the right ventricular preload.[29]

### Digoxin

Digoxin has been shown to improve cardiac output acutely in idiopathic pulmonary hypertension.[30] Its efficacy is unknown when administered chronically in patients with POPH.

### Anticoagulation

There is a favorable response with anticoagulation in other forms of PAH especially idiopathic pulmonary hypertension and chronic thromboembolic pulmonary hypertension for its ability to slow disease progression. Anticoagulation is traditionally not recommended in patients with POPH.[31] This is because of the inherent risk of hemorrhagic complications in patients with underlying liver disease and portal hypertension, especially in patients with a prior history of gastrointestinal bleeding.

### Transjugular intrahepatic portosystemic shunt

Transjugular intrahepatic portosystemic shunt (TIPS) may worsen POPH because of acute increase in preload causing increased cardiac output and mPAP. This leads to worsening right ventricular strain and dysfunction. It is not recommended in the patients with POPH.

### Specific therapy

Very limited data exist in term of medical therapy in POPH. Most of the studies are case series or case reports.

### Calcium channel blockers

Calcium channel blockers are not recommend in POPH, as they could potentially increase the hepatic venous pressure gradient and worsen portal hypertneion.[32,33]

### Prostanoids

Prostanoids have been shown to be effective in the treatment of POPH. Epoprostenol is the best studied prostanoid in POPH. Pulmonary hemodynamics associated with POPH can be improved by infusion of Epoprostenol. In moderate to severe POPH, intravenous Epoprostenol results in significant improvement (both acute and long-term) in PVR, mPAP, and cardiac output. Pulmonary hemodynamics may be improved and brain natriuretic peptide and human atrial natriuretic peptide decrease to normal levels during epoprostenol therapy. Epoprostenol may significantly improve pulmonary hemodynamics and facilitate acceptance of patients who may otherwise be denied LT as a result of POPH.[34–37] As use of Epoprostenol may potentially worsen hepatic function and cause clinical deterioration of liver disease, careful clinical follow-up is advisable. Another important reported concern with Epoprostenol is development of progressive splenomegaly with worsening thrombocytopenia;[38] however, recent data from Mayo Clinic does not demonstrate a statistically significant decrease in platelet count in a large cohort of patients with POPH treated with Epoprostenol.[39] Intravenous Iloprost, a stable prostacyclin analogue, is a valuable alternative to Epoprostenol.[40] The experience with the use of inhaled Iloprost in POPH is limited and only a few case reports are published with mixed results. A retrospective study comparing the effect of inhaled Iloprost and oral Bosentan, an endothelin-1 receptor antagonist used for three years in patients with POPH has shown that inhaled Iloprsot is significantly inferior to Bosentan in terms of survival, event free survival, exercise capacity and hemodynamics.[41]

### Endothelin receptor antagonists

Combination therapy with intravenous Iloprost and oral Bosentan, a dual endothelin-1 receptor antagonist, might extend the survival of selected patients suffering from POPH and recurrent right heart failure.[42] Bosentan has been shown to be effective in the treatment of POPH. showing clinical, functional, and hemodynamic benefits without significant hepatotoxicity in some small retrospective case series[43–46] Bosentan is probably the therapy of choice for patients with POPH as it potentially improves pulmonary as well as portal hypertension. Bosentan is potentially hepatotoxic and may cause elevation of liver enzymes in about 10% of patients with PAH. It is recommended to monitor liver functions on regular basis. No data is currently available regarding the use of other oral endothelin antagonists (for example, Sitaxsentan or Ambrisentan, which are supposedly less hepatotoxic compared to Bosentan) in this group of patients.[47]

### Phodiesterase inhibitiors

Sildenafil (Revatio) is reported to be effective in decreasing pulmonary vascular resistance.[48–49] Sildenafil is approved in a dose of 20 mg three times a day for treatment of PAH. Sildenafil was used in 14 patients with moderate to severe POPH from diverse etiologies. Eight patients were newly started on sildenafil, while six patients were already on therapy with inhaled prostanoids (Iloprost in five and Treprostinil in one). Sildenafil appeared to provide therapeutic benefit in term of decreasing the mPAP and PVR when evaluated at 3 months, but the hemodynamic benefit was not sustained after 12 months in 7 patients. The six-minute walk test continued to improve at three and 12 months.[50]

### Combination therapy

Combination vasodilator therapy has been shown to be effective in patients with idiopathic PAH.[51] Its role in POPH is not established. At present there is no convincing data to support its routine use in treating patients with POPH. Recently, case report of a single patient with severe POPH (mPAP 70 mmHg) is published who was treated with iloprost, bosentan, and sildenafil. The therapies were added sequentially beginning with inhaled iloprost which did not result in significant improvement of mPAP followed by addition of sildenafil which resulted in reduction of mPAP to 45 mmHg (after 14 days of combined therapy). The addition of bosentan for an additional month resulted in a further reduction of mPAP to 32 mmHg. There was no hepatotoxicity reported.[52]

The goal of therapy in patients with POPH, who are candidates for liver transplants, is to reduce mPAP <35 mmHg and the PVR <400 dynes/s/cm^−5^ before proceeding to liver transplant. Perioperative mortality in patients with mean PAP >35 mmHg is significantly higher compared to those with mPAP < 35 mmHg.[53]

## Conclusion

POPH is a type of PAH associated with portal hypertension. The proposed pathophysiology includes hyperdynamic pulmonary circulation leading to shear stress of pulmonary vasculature causing obstructive vasculopathy and increased pulmonary resistance. The diagnosis of POPH is suggested by TTE and is confirmed by right heart catheterization. Mortality in advance liver disease in the presence of pulmonary hypertension is high. Every effort should be made to treat these patients with vasodilator therapy to reduce PAP and PVR, and ultimately improve the right heart function. Treatment with a combined approach of pulmonary arterial vasomodulator therapy and liver transplant may improve long term survival in patient with POPH. Randomized controlled trials are needed to determine the future direction in management of POPH.

## References

[1] Mandell MS, Groves BM (1996). Pulmonary hypertension in chronic liver disease. Clin Chest Med.

[2] Budhiraja R, Hassoun PM (2003). Portopulmonary hypertension: a tale of two circulations. Chest.

[3] Castro M, Krowka MJ, Schroeder DR, Beck KC, Plevak DJ, Rettke SR (1996). Frequency and clinical implications of mincreased pulmonary artery pressures in liver transplant patients. Mayo Clinic Proc.

[4] Hadengue A, Benhayoun MK, Lebrec D, Benhamou JP (1991). Pulmonary hypertension complicating portal hypertension: prevalence and relation to splachnic hemodynamics. Gastroenterology.

[5] Humbert M, Sitbon O, Chaouat A, Bertocchi M, Habib G, Gressin V (2006). Pulmonary arterial hypertension in France: results from a national registry. Am Rev Respir Crit Care Med.

[6] Passarella M, Fallon MB, Kawut SM (2006). Portopulmonary hypertension. Clin Liver Dis.

[7] Kawut SM, Krowka MJ, Trotter JF, Roberts KE, Benza RL, Badesch DB (2008). Clinical risk factors for portopulmonary hypertension. Hepatology.

[8] Roberts KE, Fallon MB, Krowka MJ, Brown RS, Trotter JF, Peter I (2009). Genetic risk fact for portopulmonary hypertension in patients with advanced liver disease. Am J Respir Crit Care Med.

[9] Swanson KL, Wiesner RH, Nyberg SL, Rosen CB, Krowka MJ (2008). Survival in portopulmonary hypertension: Mayo Clinic experience categorized by treatment subgroups. Am J Transpl.

[10] Hadengue A, Benhayoun MK, Lebrec D, Benhamou JP (1991). Pulmonary hypertension complicating portal hypertension: prevalence and relation to splachnic hemodynamics. Gastroenterology.

[11] Gaine SP, Rubin LJ (1998). Primary pulmonary hypertension. Lancet.

[12] Pellicelli AM (2004). Cytokines in portopulmonary hypertension. Gut.

[13] Edwards BS, Weir EK, Edwards WD, Ludwig J, Dykoski RK, Edwards JE (1987). Coexistent pulmonary and portal hypertension: morphologic and clinical features. J Am Coll Cardiol.

[14] Jamison BM, Michel RP (1995). Different distribution of plexiform lesions in primary and secondary pulmonary hypertension. Hum Pathol.

[15] Rodriguez-Roisin R, Krowka MJ, Herve P, Fallon MB (2004). Pulmonary-hepatic vascular disorders (PHD). Eur Respir J.

[16] Hoeper MM, Krowka MJ, Strassburg CP (2004). Portopulmonary hyper- tension and hepatopulmonary syndrome. Lancet.

[17] Porres-Aguilar M, Anaya-Ayala JE, Porres-Munoz M, Bracamontes F (2007). Chronic thromboembolic pulmonary hypertension. Cir.

[18] Krowka MJ (2000). Pulmonary hypertension. Mayo Clin Proc.

[19] Swanson KL, Krowka MJ (2002). Arterial oxygenation associated with portopulmonary hypertension. Chest.

[20] Murray KF, Carithers RL (2005). AASLD practice guidelines: evaluation of patients for liver transplantation. Hepatology.

[21] Krowka MJ, Swanson KL, Frantz RP, McGoon MD, Wiesner RH (2006). Portopulmonary hypertension: results from a 10-year screening algorithm. Hepatology.

[22] Rodriguez-Roisin R, Krowka MJ, Herve P, Fallon MB (2004). Pulmonary-Hepatic vascular Disorders (PHD). Eur Respir J.

[23] Ricci GL, Melgosa MT, Burgos F, Valera JL, Pizarro S, Roca J Assessment of acute pulmonary vascular reactivity in portopulmonary hypertension.

[24] Krowka MJ, Plevak DJ, Findlay JY, Rosen CB, Wiesner RH, Krom RA (2000). Pulmonary hemodynamics and perioperative cardiopulmonary mortality in patients with portopulmonary hypertension undergoing liver transplantation. Liver Transpl.

[25] Ramsay MA, Simpson BR, Nguyen AT, Ramsay KJ, East C, Klintmalm GB (1997). Severe pulmonary hypertension in liver transplant candidates. Liver Transpl Surg.

[26] Krowka MJ, Mandell MS, Ramsay MA, Kawut SM, Fallon MB, Manzarbeitia C (2004). Hepatopulmonary syndrome and portopulmonary hypertension: a report of the multicenter liver transplant database. Liver Transpl.

[27] Starkel P, Vera A, Gunson B, Mutimer D (2002). Outcome of liver transplantation for patients with pulmonary hypertension. Liver Transpl.

[28] Porres-Aguilar M, Zuckerman MJ, Figueroa-Casas JB, Krowka MJ (2008). Portopulmonary hypertension: state of the art. Annals of Hepatology.

[29] Hoeper MM, Krowka MJ, Strassburg CP (2004). Portopulmonary hypertension and hepatopulmonary syndrome. Lancet.

[30] Rich S, Seidlitz M, Dodin E, Osimani D, Judd D, Genthner D (1998). The short-term effects of digoxin in patients with right ventricular dysfunction from pulmonary hypertension. Chest.

[31] Rodriguez-Roisin R, Krowka MJ, Herve P, Fallon MB (2004). Pulmonary- hepatic vascular disorders (PHD). Eur Respir J.

[32] Ota K, Shijo H, Kokawa H, Kubara K, Kim T, Akiyoshi N (1995). Effects of nifedipine on hepatic venous pressure gradient and portal vein blood flow in patients with cirrhosis. J Gastroenterol Hepatol.

[33] Navasa M, Bosch J, Reichen J, Bru C, Mastai R, Zysset T (1988). Effects of verapamil on hepatic and systemic hemodynamics and liver function in patients with cirrhosis and portal hypertension. Hepatology.

[34] Krowka MJ, Frantz RP, McGoon MD, Severson C, Plevak DJ, Weisner RH (1999). Improvement in pulmonary hemodynamics during intravenous epoprostenol (prostacyclin): a study of 15 patients with moderate to severe portopulmonary hypertension. Hepatology.

[35] Fix OK, Bass NM, De Marco T, Merriman RB (2007). Long-term follow- up of portopulmonary hypertension: effect of treatment with epoprostenol. Liver Transpl.

[36] Tan HP, Markowitz JS, Montgomery RA, Merritt WT, Klein AS, Thuluvath PL (2001). Liver transplantation in patients with severe portopulmonary hypertension treated with preoperative chronic intravenous epoprostenol. Liver Transpl.

[37] Kuo PC, Johnson LB, Plotkin JS, Howell CD, Bartlett ST, Rubin LJ (1997). Continuous intravenous infusion of epoprostenol for the treatment of portopulmonary hypertension. Transplantation.

[38] Findlay JY, Plevak DJ, Krowka MJ, Sack EM, Porayko MK (1999). Progressive splenomegaly after epoprostenol therapy in portopulmonary hypertension. Liver Transpl Surg.

[39] Golbin JM, Krowka MJ (2006). Effect of prostacyclins on thrombocytopenia in portopulmonary hypertension. Data presented at the 2006 Pulmonary Hypertension Association International Conference.

[40] Minder S, Fischler M, Muellhaupt B, Zalunardo MP, Jenni R, Clavien PA (2004). Intravenous iloprost bridging to orthotopic liver transplantation in portopulmonary hypertension. Eur Respir J.

[41] Halank M, Kolditz M, Miehlke S, Schiemanck S, Schmeisser A, Hoeffken G (2005). Combination therapy for portopulmonary hypertension with intravenous iloprost and oral bosentan. Wien Med Wochenschr.

[42] Hoeper MM, Seyfarth HJ, Hoeffken G, Wirtz H, Spiekerkoetter E, Pletz MW (2007). Experience with inhaled iloprost and bosentan in portopulmonary hypertension. Eur Respir J.

[43] Kuntzen C, Guelberg V, Gerbes AL (2005). Use of a mixed receptor antagonist in portopulmonary hypertension: a safe and effective therapy?. Gastroenterology.

[44] Hinterhuber L, Graziadei IW, Kahler CM, Jaschke W, Vogel W (2004). Endothelin-receptor antagonist treatment of portopulmonary hypertension. Clin Gastroenterol Hepatol.

[45] Hoeper MM, Halank M, Marx C, Hoeffken G, Seyfarth HJ, Schauer J (2005). Bosentan therapy for portopulmonary hypertension. Eur Respir J.

[46] Halank M, Miehke S, Hoeffken G, Schmeisser A, Schulze M, Strasser RH (2004). Use of oral endothelin receptor antagonist bosentan in the treatment of portopulmonary hypertension. Transplantation.

[47] Idrees MM, Al-Hajjaj M, Khan J, Al-Hazmi M, Alanezi M, Saleemi S (2008). Saudi guidelines on diagnosis and treatment of pulmonary arterial hypertension. Ann Thorac Med.

[48] Chua R, Keogh A, Miyashita M (2005). Novel use of sildenafil in the treatment of portopulmonary hypertension. J Heart Lung Transplant.

[49] Makisalo H, Koivusalo A, Vakkuri A, Hockerstedt K (2004). Sildenafil for portopulmonary hypertension in a patient undergoing liver transplantation. Liver Transpl.

[50] Reichenberger F, Voswinckel R, Steveling E, Enke B, Kreckel A, Olschewski H (2006). Sildenafil treatment for portopulmonary hypertension. Eur Respir J.

[51] Murali S (2005). Combination therapy: The Future of medical management for PAH. Medscape Cardiol.

[52] Austin MJ, McDougall NI, Wendon JA, Sizer E, Knisely AS, Rela M (2008). Safety and efficacy of combined use of sildenafil, bosentan, and iloprost before and after liver transplantation in severe portopulmonary hypertension. Liver Transpl.

[53] Krowka MJ, Plevak DJ, Findlay JY, Rosen CB, Weisner RH, Krom RA (2000). Pulmonary hemodynamics and perioperative cardiopulmonary related mortality in patients with portopulmonary hypertension undergoing liver transplantation. Liver Transpl.

